# Vorinostat Eliminates Multicellular Resistance of Mesothelioma 3D Spheroids via Restoration of Noxa Expression

**DOI:** 10.1371/journal.pone.0052753

**Published:** 2012-12-26

**Authors:** Dario Barbone, Priscilla Cheung, Sailaja Battula, Sara Busacca, Steven G. Gray, Daniel B. Longley, Raphael Bueno, David J. Sugarbaker, Dean A. Fennell, V. Courtney Broaddus

**Affiliations:** 1 Lung Biology Center, San Francisco General Hospital, University of California San Francisco, San Francisco, California, United States of America; 2 Department of Cancer Studies and Molecular Medicine, University of Leicester, Leicester, United Kingdom; 3 Department of Cardiothoracic Surgery, St James's Hospital, Dublin, Ireland; 4 Centre for Cancer Research and Cell Biology, The Queen's University of Belfast, Belfast, Northern Ireland, United Kingdom; 5 Department of Thoracic Surgery, Brigham and Women's Hospital, Harvard Medical School, Boston, Massachusetts, United States of America; The Moffitt Cancer Center & Research Institute, United States of America

## Abstract

When grown in 3D cultures as spheroids, mesothelioma cells acquire a multicellular resistance to apoptosis that resembles that of solid tumors. We have previously found that resistance to the proteasome inhibitor bortezomib in 3D can be explained by a lack of upregulation of Noxa, the pro-apoptotic BH3 sensitizer that acts via displacement of the Bak/Bax-activator BH3-only protein, Bim. We hypothesized that the histone deacetylase inhibitor vorinostat might reverse this block to Noxa upregulation in 3D. Indeed, we found that vorinostat effectively restored upregulation of Noxa protein and message and abolished multicellular resistance to bortezomib in the 3D spheroids. The ability of vorinostat to reverse resistance was ablated by knockdown of Noxa or Bim, confirming the essential role of the Noxa/Bim axis in the response to vorinostat. Addition of vorinostat similarly increased the apoptotic response to bortezomib in another 3D model, the tumor fragment spheroid, which is grown from human mesothelioma *ex vivo*. In addition to its benefit when used with bortezomib, vorinostat also enhanced the response to cisplatin plus pemetrexed, as shown in both 3D models. Our results using clinically relevant 3D models show that the manipulation of the core apoptotic repertoire may improve the chemosensitivity of mesothelioma. Whereas neither vorinostat nor bortezomib alone has been clinically effective in mesothelioma, vorinostat may undermine chemoresistance to bortezomib and to other therapies thereby providing a rationale for combinatorial strategies.

## Introduction

Solid tumors such as mesothelioma are characterized by a stubborn resistance to chemotherapy, mostly due to resistance to apoptosis. We have explored the mechanisms of this apoptotic resistance in 3D spheroids, in which cancer cells acquire a broad multicellular resistance to apoptosis that may replicate some of the chemoresistance seen in human solid tumors. Indeed, we have been encouraged that several of our findings in 3D multicellular spheroids have been replicated in our studies of human mesothelioma tumor tissue grown ex vivo [1], [2].

Based on our prior studies in both mesothelioma and in lung cancer, the apoptotic resistance seen in 3D spheroids appeared to be mediated by alterations in the Bcl-2 family [1], [3]. In mesothelioma cells grown in 3D, the baseline expression of the Bcl-2 family members and the response of the Bcl-2 proteins to pro-apoptotic stress differed significantly from that in the same cells in 2D. One intriguing finding was that the Bak/Bax-activator BH3-protein Bim was elevated in 3D spheroids compared to 2D monolayers. Moreover, the bortezomib-mediated increase in Noxa, the BH3 protein that displaces Bim and thereby mediates apoptosis, failed to increase. Although we could induce apoptosis in these spheroids by displacing Bim using a BH3-mimetic (ABT-737) or by replacing Noxa with a cell permeable R8-Noxa BH3 peptide, we sought a clinically relevant mechanism by which we could restore the cellular upregulation of Noxa. For this, we tested a histone deacetylase inhibitor, vorinostat, an agent known to restore expression of several pro-apoptotic genes, including Noxa, in resistant settings [4], [5].

Vorinostat has been shown to induce apoptosis in many *in vitro* studies of solid tumors, including mesothelioma [5], [6], largely by unwinding condensed chromatin and allowing transcription of epigenetically silenced genes. In addition, HDAC inhibitors such as vorinostat have modulatory effects on other HDAC substrates, such as chaperones [7], and can activate immune and inflammatory processes [8]. Unfortunately, despite these promising features, in a recent large clinical trial of patients with mesothelioma, vorinostat proved to be ineffective [*unpublished*, http://goo.gl/dZCFx]. Similarly, after multiple promising *in vitro* studies, bortezomib has also been reported to be ineffective in patients with mesothelioma [9]. We, as other groups in the mesothelioma research field, believe that studying chemoresistance in 3D can reveal novel mechanisms [10]–[12]. Hence we wished to use our resistant 3D models to investigate the activity of each agent alone and the activity of the two agents in combination. If vorinostat could restore the ability of bortezomib to upregulate Noxa in our 3D models, then these two agents could potentially be useful in combination in the clinical setting.

In this report, we show that the histone deacetylase inhibitor vorinostat eliminated the apoptotic resistance to bortezomib acquired by 3D spheroids. This was shown to be due to the ability of vorinostat to restore bortezomib-induced upregulation of Noxa protein expression in the 3D spheroids. In both 3D models, multicellular spheroids and tumor fragment spheroids grown from mesothelioma tumor *ex vivo*, vorinostat had little apparent apoptotic effect by itself but acted to enhance the apoptosis induced by bortezomib. In both spheroid models, vorinostat also amplified the apoptosis induced by cisplatin plus pemetrexed, suggesting that vorinostat could improve the response to a variety of therapeutic approaches.

## Materials and Methods

### Cell cultures and reagents

The human mesothelioma cell lines M28 and VAMT were obtained from Dr. Brenda Gerwin [13], [14]. The human mesothelioma cell lines REN and SARC (MesoSA1) were obtained from Dr. Steven Albelda [15] and from Dr. Alice Boylan [16], respectively. All cells were cultured in DMEM supplemented with 10% FBS and 100 IU/mL penicillin/streptomycin in a 37°C humidified incubator with 5% CO_2_ (full DMEM).

A standard dose of bortezomib (100 nM) was used for short-term experiments such as western blots and qRT-PCR. A lower dose (25 nM) was used in all apoptotic assays at 24 h. Vorinostat (Zolinza®) was from Selleck Chem (Boston, MA). Bortezomib (Velcade®) was from Millennium Pharmaceuticals (Cambridge MA). Cisplatin and pemetrexed were obtained from the UCSF Pharmacy at Mt. Zion Cancer Center.

### Generation and treatment of spheroids

*Multicellular spheroids* were generated in non-adsorbent round-bottomed 96-well plates, as described [17]. The 96-well plates were coated with a 1∶24 dilution of polyHEMA (120 mg/ml) (#P3932 Sigma-Aldrich, St. Louis, MO) in 95% ethanol and dried at 37°C for 24 h. Before use, plates were sterilized by UV light for 30 min. For generation of multicellular spheroids, 10^4^ cells were added into each well of polyHEMA-coated 96-well plate. The plates were briefly spun for 5 min at 800 rpm and then placed in a 37°C humidified incubator with 5% CO_2_ for 48 h. For generation of monolayers, 180,000 cells were added into each well of 6-well plates. Bright field images were taken with a Zeiss Invertoskop 40C.

*Tumor Fragment Spheroids* were generated as previously described [2] from six tumor samples obtained from extrapleural pneumonectomy (EPP) or pleurectomy procedures performed by D.J.S. and R.B. at Brigham and Women's Hospital in Boston, MA USA. A part of the tumor was fixed in 10% formalin (Fisher Scientific, Fair Lawn, NJ) and embedded in paraffin. For spheroid culture, tumor tissue was diced finely with scalpels to pieces smaller than 1 mm in diameter that were suspended in medium in 10-cm plates coated with 0.8% agar (Agar Noble, #A5431 Sigma-Aldrich, St. Louis, MO) in full DMEM. The volume of overlay media was 15 ml, and half the volume of the overlay media was changed twice a week. The agar-coated plates were regularly observed using an inverted phase microscope during the incubation period, up to 4 weeks. Spheroids were collected at different time points, treated as described in figure legends, fixed in 10% formalin, and embedded in paraffin for immunostaining.

#### Treatment

Before treatment, 18 multicellular spheroids (or 20–30 tumor fragment spheroids) were transferred to each well of a polyHEMA-coated 24-well plate to match the numbers of cells plated as monolayers (180,000 cells per well). The spheroids and monolayers were treated with apoptotic agents in full DMEM with or without inhibitors (and the appropriate DMSO vehicle control) for 24 h.

### Immunoblotting

After treatment, monolayers and spheroids were lysed in boiling lysis buffer (2.5% SDS, Tris-HCl 250 mM pH 7.4). The concentration of total protein was evaluated with a colorimetric assay (DC protein assay from BioRad, Hercules, CA). 50 µg of cell lysates were loaded in reducing conditions (0.2 M Tris, pH 6.8, 5% SDS, 3% glycerol, 0.01% bromophenol blue and 200 mM DTT). After separation in SDS-PAGE (5 to 15% acrylamide) and transfer to PVDF (Immobilon, Millipore, Billerica, MA), membranes were blocked with a protein-free TBS blocking buffer (Pierce, Rockford, IL) and gently agitated with antibodies diluted in 5% non-fat dry milk or 5% BSA, as appropriate, at 4°C overnight. Secondary antibodies were from Amersham (Piscataway, NJ). Chemiluminescence was detected with the enhanced SuperSignal West Pico Substrate (Pierce, Rockford, IL) with a Biospectrum Imaging System (UVP, Upland, CA). The antibody against Bim (#559685) was from BD Pharmingen (San Jose, CA) and the Noxa antibody was from Calbiochem (#OP180). The α-tubulin antibody (#T-6074) was from Sigma-Aldrich (St. Louis, MO). Densitometry of the bands was analyzed with the VisionWorksLS software (UVP, Upland, CA). Total raw pixel density of each band was normalized to its respective tubulin band and expressed as a ratio.

### Co-immunoprecipitation

Spheroids (100 for each condition) were treated as indicated and lysed in ice-cold CHAPS buffer (1% (w/v) CHAPS, 10 mm HEPES pH 7.4, 150 mm NaCl) plus protease inhibitor cocktail and EDTA (Pierce, Rockford, IL) for 30 min. Lysates were then centrifuged at 15,000 rpm for 15 min at 4°C. 500 µg of the cleared lysates were pre-incubated at 4°C for 1 h with 50 µl of a 50∶50 slurry of CHAPS buffer and Sepharose Prot G (Amersham, Piscataway, NJ). Pre-cleared lysates were then incubated with the Mcl-1 antibody (Santa Cruz, SC-189, 2 µg per condition) for 1 h at 4°C. 50 µl of a 50∶50 slurry of CHAPS buffer and Sepharose Prot G (Amersham, Piscataway, NJ) were added, and lysates were incubated overnight at 4°C to capture the immune complexes. Beads were then washed five times with ice-cold CHAPS buffer and bound proteins were eluted with 50 µl of a low pH elution buffer (#1858606, Pierce, Rockford, IL). Recovered lysates were neutralized by addition of 5 µl of Tris-HCl 1.5 M pH 8.8 and boiled for 5 min with Laemmli Buffer (glycerol 10%, bromophenol blue 1%, dithiothreitol (DTT) 1 M, SDS 10%, Tris-Cl 1 M, pH 6.8). Immunoblotting was performed as described above.

### RNA interference

Cells (4×10^6^) were pelleted and resuspended in 100 µl of nucleofection buffer (solution T, Amaxa Biosystems, Cologne, Germany) with 1.5 µg of the appropriate siRNA duplex (Ambion, Austin, TX). This suspension was transferred to a sterile cuvette and nucleofected using program T-20 on a Nucleofector II device (Amaxa Biosystems, Cologne, Germany). After 30 min recovery in complete DMEM medium, the cells were plated and allowed to grow for 24 h. Cells were then trypsinized, counted and plated as monolayers and spheroids for 24 h, and exposed to apoptotic stimuli. The siRNA sequences were: Bim (ACUUACAUCAGAAGGUUGCtt), Noxa (GAAAUGUGUCAAUAAUUACtt), non-targeting control (GCAACCUUCUGAUGUAAGUtt). Cells transfected with siRNA were also allowed to form into spheroids, treated with bortezomib and subjected to immunoblotting to determine if Noxa and Bim were effectively ablated.

### Measurement of apoptosis in multicellular spheroids

#### Nuclear condensation

Apoptosis was quantitated by counting cells with nuclear condensation. Monolayers and spheroids were disaggregated by exposure to trypsin for the same period of time, washed with ice-cold PBS, and then fixed with 2.5% glutaraldehyde (Sigma-Aldrich, St. Louis, MO). The cells were then stained with 8 µg/ml of Hoechst 33342 (Molecular Probes, Invitrogen, Carlsbad, CA) and placed on slides. Apoptosis was quantitated by counting cells with distinctive signs of nuclear condensation and expressed as a % of the total cells. For each condition, at least 300 cells were counted in triplicate by investigators blinded to the experimental conditions.

#### Caspase activation

Apoptosis was also measured by an assay of caspase activation, by use of a Caspase-Glo® 3/7 assay kit (Promega, Madison WI). In this assay, the level of caspase activity is measured by the caspase-3/7 mediated cleavage of a proluminescent caspase-3/7 substrate, which releases a substrate for luciferase and thereby produces light. Briefly, spheroids were treated as indicated in the polyHEMA-coated 96 wells in which they were grown (200 µl final volume). One spheroid was used for each condition; studies were performed in quadruplicate. After treatment, plates were spun at 400 g for 10 min RT; 100 µl of supernatant was carefully removed without disturbing the pellet and 100 µl of complete CaspaseGlo® 3/7 reagent was added to each well. Plates were gently mixed using a plate shaker at 300–500 rpm for 1 min and then incubated at RT away from light for 1 h. Sample luminescence was measured in a plate-reading luminometer (Perkin Elmer, Waltham, MA).

### Measurement of apoptosis in tumor fragment spheroids

Measurement of apoptosis in this complex tissue required dual immunostaining to identify the mesothelioma cells and thus to localize the cleaved caspase 3 characteristic of apoptosis to the mesothelioma cells, as we have previously reported [18]. Following treatment, tumor fragment spheroids were collected, fixed in 10% buffered formalin in PBS overnight at 4°C and embedded in 3% agar in PBS. The agar pellets containing the tumor fragment spheroids were further embedded in paraffin blocks by the UCSF pathology core at SFGH. For immunostaining, 5 µm paraffin sections were deparaffinized with Xylene (2×5 min), 100% EtOH (2×2 min), 95% EtOH (2×2 min), 70% EtOH (2×2 min), 50% EtOH (1×2 min) and ddH20 (2×2 min). Endogenous peroxidases were blocked with a solution of 250 ml MeOH +5 ml 30% H_2_0_2_ for 20 min. Antigens were retrieved in citrate buffer (Citra, BioGenex; #HK087-5K) in a microwave oven set to high for 7–8 min. Sections were blocked with 5% normal goat serum, 2.5% BSA in PBS for 30–45 min in a humidified chamber at RT. Primary antibodies for cleaved caspase 3 (1∶100, Chemicon; #AB3623) and pan-cytokeratin (1∶100, Progen, Heidelberg, Germany; clone GP14) were incubated in a humidified chamber at 4°C overnight. The secondary antibodies donkey anti-rabbit AlexaFluor 488 (Pierce, Rockford, IL; #31821) and anti-guinea pig AlexaFluor 633 (Invitrogen, Carlsbad, CA; #A-21105), both 1∶200, were incubated for 30–45 min at RT in a humidified chamber. Slides were washed 3 times in PBS for 3 min and mounted with Vectashield. In a blinded fashion, the investigators examined images of doubly stained cells in the tumor fragment spheroids. Apoptotic mesothelioma cells were considered to be cells with merged red (pan-cytokeratin) and green (cleaved caspase 3) and were expressed as a percentage of the total number of mesothelioma cells (red). For each condition, 3–10 spheroids were counted until a total of 300 mesothelioma cells were visualized.

### Immunohistochemistry of tumor fragment spheroids

New sections were cut from the paraffin blocks of the tumor fragment spheroids used for the apoptosis studies described above and were probed for expression of Bim and Noxa protein by immunohistochemistry. 5 µm paraffin sections were stained using a Bim antibody (#559685BD, Pharmingen, San Jose, CA - 1∶200, 2 h, 4°C) and a Noxa antibody (#OP-180, Calbiochem – 1∶200, 2 h, 4°C) and visualized with a HRP/DAB Envision plus Kit (#K4010, Dako, Carpinteria, CA). Hematoxylin was used as a counterstain.

### Real-Time quantitative polymerase chain reaction

M28 or REN cells (10^6^) were plated as monolayers or spheroids (100 spheroids – 10^4^ cells/spheroid) and RNA was extracted with an RNeasy Kit following manufacturer's instructions (Qiagen, Valencia, CA). Extracted total RNA was quantified with a NanoDrop 2000c (NanoDrop, Wilmington, DE) and 2 µg of total RNA was retro-transcribed with a Superscript® VILO cDNA Synthesis Kit (Life Technologies, Carlsbad, CA). A TaqMan® Gene Expression Analysis Assay (Life Technologies, Carlsbad, CA) was performed according to manufacturer's instructions in a ABI 7000 thermal cycler (Life Technologies, Carlsbad, CA). Taqman probes for Noxa (Hs00560402_m1), Bim (Hs01076940_m1), Bmf (Hs00372937_m1) and the control beta glucuronidase (GUSB 4333767T) were from Applied Biosystems (Life Technologies, Carlsbad, CA). Calculations for determining the relative levels of gene expression were made from triplicate measurements of the target gene, with normalization to *GUSB* in the samples, using the cycle threshold (Ct) method and the 2^−ΔΔct^ equation [19].

### Statistical analysis

Data are expressed as mean ± one standard deviation of at least three different experiments. Statistical significance was evaluated by one or two-way analysis of variance, and Tukey's test was performed to detect where the differences lay (GraphPad Prism version 4.0, GraphPad Software, Inc.). A p value less than 0.05 was considered significant.

## Results

### Vorinostat eliminates multicellular resistance of spheroids

**Figure 1:**
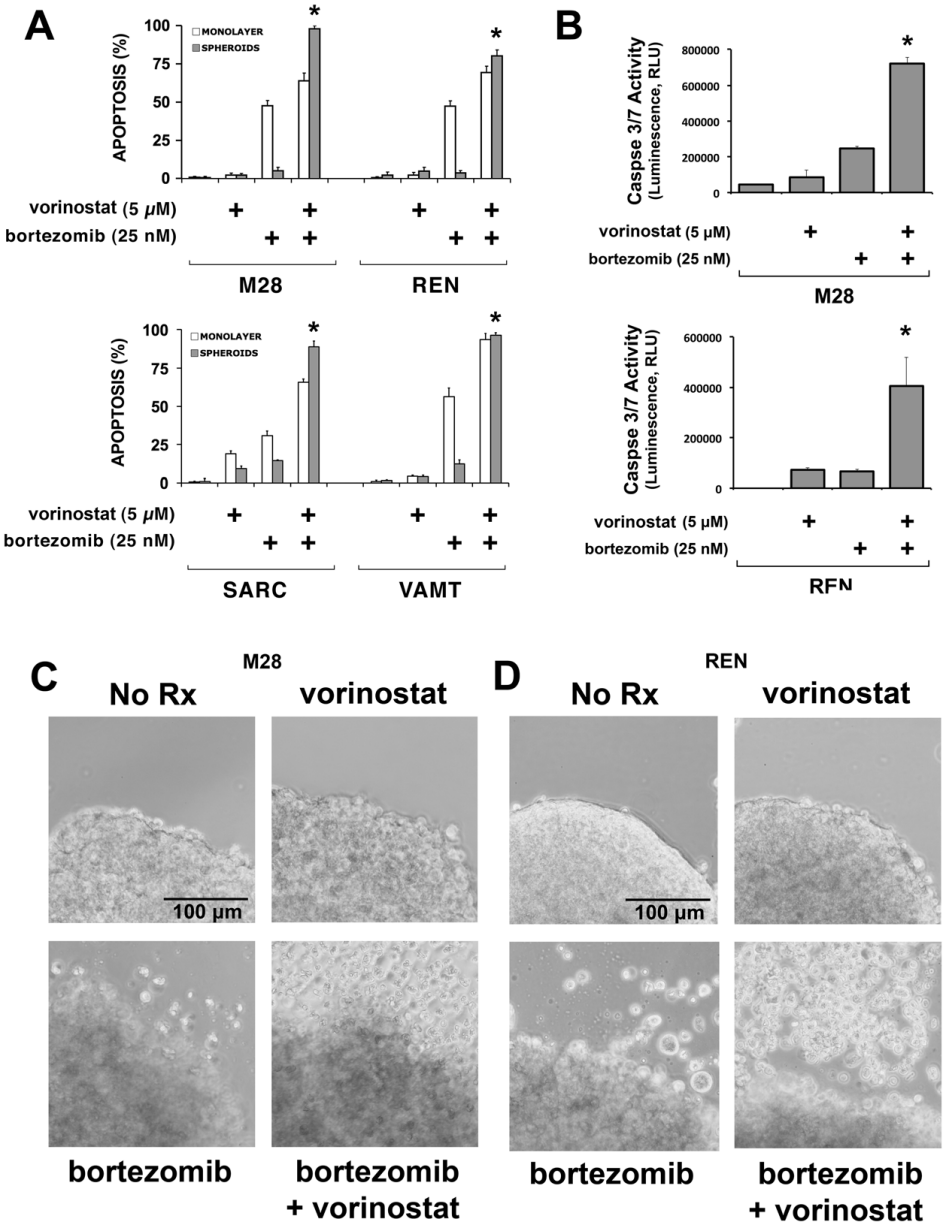
Vorinostat abolishes multicellular resistance of spheroids. (**A**) Four mesothelioma cell lines (M28, REN, SARC and VAMT) were grown as monolayers and spheroids and treated with bortezomib (25 nM), vorinostat (5 µM) or their combination for 24 h. Apoptosis was measured by analysis of nuclear condensation in Hoechst-stained cells. Spheroids grown from all the cell lines acquired multicellular resistance to bortezomib, a resistance that was effectively eliminated by the addition of vorinostat. Both vorinostat and bortezomib induced little apoptosis in spheroids when given alone. (* p<0.05 as compared to bortezomib alone, n = 3) (**B**) Apoptosis was confirmed by CaspaseGlo 3/7 assay in M28 and REN spheroids, treated with either bortezomib (25 nM), vorinostat (5 µM) or their combination for 24 h. Vorinostat had no effect on spheroids but increased their apoptotic response to bortezomib (* p<0.05 as compared to bortezomib or vorinostat alone, n = 3) (**C–D**) Bright field 10× images of the edge of M28 and REN spheroids treated with vorinostat, bortezomib or the combination for 24 h. Whereas vorinostat or bortezomib alone induced little or no morphologic change, the combination potently increased the number of cells detaching from the spheroids and floating in the medium. (Focus was adjusted in the lower panels to visualize some of these floating cells).

Four mesothelioma cell lines (M28, REN, SARC and VAMT) were grown as 2D monolayers or 3D spheroids and treated with the proteasome inhibitor bortezomib, As expected, all cell lines grown as 3D spheroids demonstrated multicellular apoptotic resistance to bortezomib. Remarkably, in all four lines, when vorinostat was added in combination with bortezomib, vorinostat effectively abolished the multicellular resistance to bortezomib, as shown by the restoration of apoptosis in the cells in 3D to the same or greater level as that seen in 2D (**Figure 1A–D**). When given alone, however, vorinostat did not induce apoptosis in either monolayers or spheroids (**Figure 1A–D**). A second assay of apoptosis, measurement of caspase activation, confirmed that vorinostat alone had no effect but, when given in combination with bortezomib, effectively increased apoptosis of cells in 3D spheroids (**Figure 1B**). Vorinostat alone had no evident effect on the morphology of spheroids (**Figure 1C–D**) however, when given with bortezomib, vorinostat clearly amplified the morphologic alterations seen with bortezomib. The floating cells, shown as detached from spheroids, were all found to be non-viable (*data not shown*). As seen with bortezomib, vorinostat also amplified the apoptotic response to cisplatin plus pemetrexed, the standard treatment used for patients with mesothelioma [20] (**Figure S1**).

### Vorinostat restores bortezomib-induced increase of Noxa protein and mRNA in 3D

Our previous studies identified a lack of Noxa upregulation in 3D mesothelioma spheroids as a cause of multicellular resistance to bortezomib [1]. To confirm if vorinostat restored Noxa upregulation, we analysed its protein and mRNA expression in M28 and REN cells treated with bortezomib, with or without vorinostat, and harvested at 4–6 h before the onset of apoptosis.

**Figure 2:**
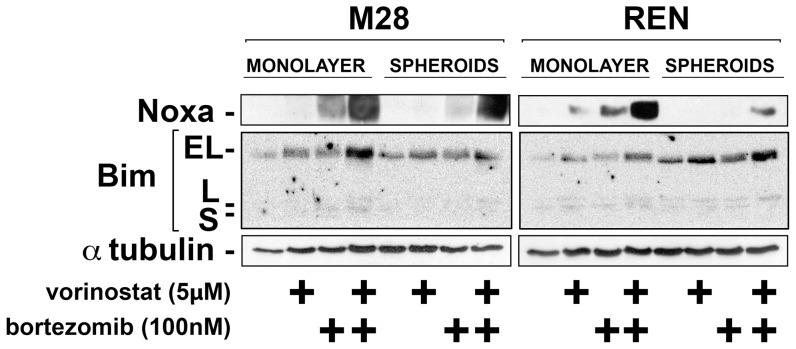
Vorinostat restores the upregulation of Noxa induced by bortezomib in spheroids. M28 and REN monolayers and spheroids were treated with bortezomib (100 nM), vorinostat (5 µM) or the combination for 6 h. Cells were then lysed and Noxa and Bim levels were analyzed by immunoblot. In both M28 and REN cells grown as monolayers, bortezomib increased levels of Noxa protein; however, in the same cells grown as spheroids, the bortezomib-induced increase in Noxa was blunted or absent. With the addition of vorinostat, bortezomib now increased Noxa protein in the spheroids to levels comparable to or greater than those in bortezomib-treated monolayers. Of note, vorinostat alone had little or no effect on Noxa protein levels. Vorinostat alone and particularly in combination with bortezomib increased the levels of Bim protein. (Densitometry of band intensities relative to tubulin is shown in **Figure S2A and S2B**).

Upon treatment with bortezomib, Noxa protein expression increased in the cells in 2D; however, as previously observed, this increase was either not seen or was much reduced in 3D spheroids (**Figure 2** **–** densitometry analysis in **Figure S2A and S2B**). When used alone, vorinostat had little effect on Noxa protein expression; however, when used in combination with bortezomib, vorinostat significantly increased Noxa expression in both 2D and 3D. In the 3D spheroids, Noxa either reached the level seen after bortezomib alone in 2D (REN) or exceeded it (M28). Because Noxa is reported to act via the displacement of Bim from anti-apoptotic reservoirs, we also investigated changes in Bim expression. Vorinostat alone and particularly in combination with bortezomib increased Bim in mesothelioma cells in both 2D and 3D. Vorinostat also increased expression of Noxa and Bim in spheroids when used in combination with cisplatin plus pemetrexed (**Figure S3**).

**Figure 3:**
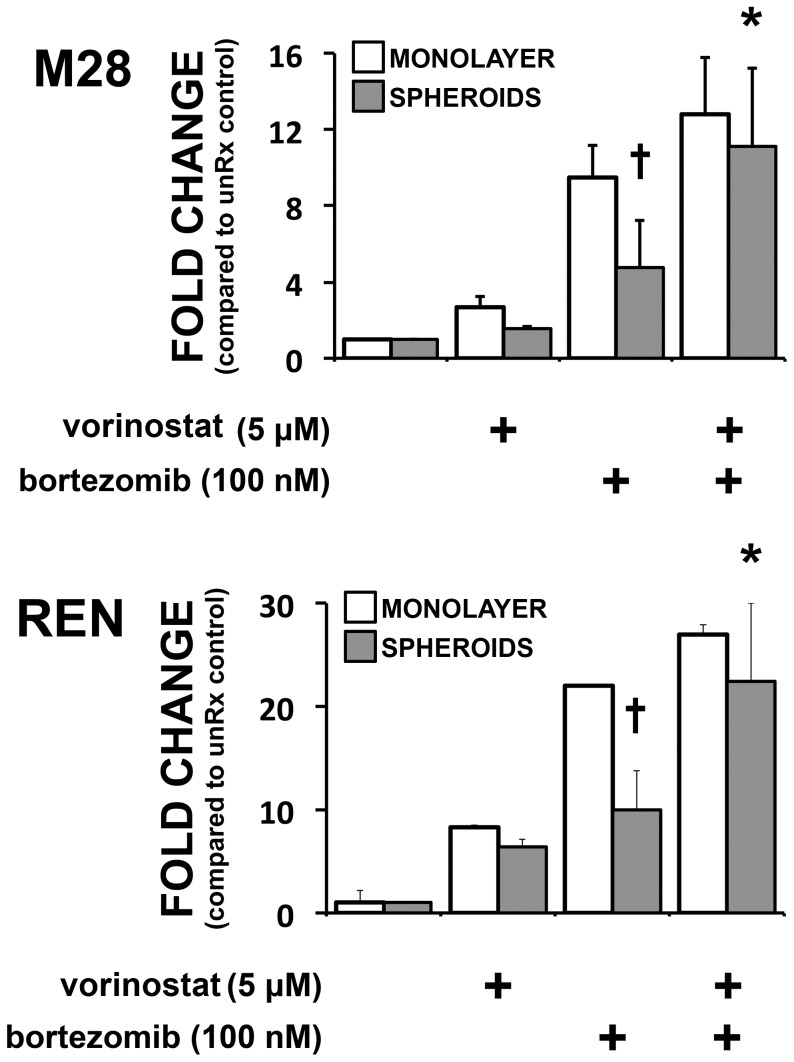
Vorinostat restores Noxa mRNA of spheroids to the level of monolayers. M28 and REN monolayers and spheroids were treated with bortezomib (100 nM), vorinostat (5 µM) or their combination for 4 h and Noxa mRNA was measured by qRT-PCR. After treatment with bortezomib, Noxa mRNA increased significantly in monolayers but was significantly lower in spheroids. The addition of vorinostat to bortezomib led to an increase in Noxa mRNA levels in spheroids to the same level as in monolayers. (* p<0.05, significant increase compared to bortezomib alone; † p<0.05, significantly less than in the monolayer exposed to same treatment; n = 3).

We examined the transcription of Noxa message to determine whether it was suppressed in 3D and whether vorinostat restored transcription. Indeed, after bortezomib, Noxa message increased in cells grown in 2D but did not increase significantly in cells in 3D (**Figure 3**). Vorinostat appeared to remove the suppression of Noxa transcription in 3D; after the combination of vorinostat with bortezomib, Noxa message increased to the same extent in cells in 3D as in 2D.

In addition to Noxa and Bim, Bmf has been reported to respond to vorinostat (10) and possibly to activate Bax/Bak directly (11). However, we found that Bim and Bmf message increased no more than 2–3 fold whereas Noxa message increased by 10–20 fold following the combination of vorinostat plus bortezomib (**Figure S4**).

### Vorinostat eliminates multicellular resistance via Noxa/Bim

**Figure 4:**
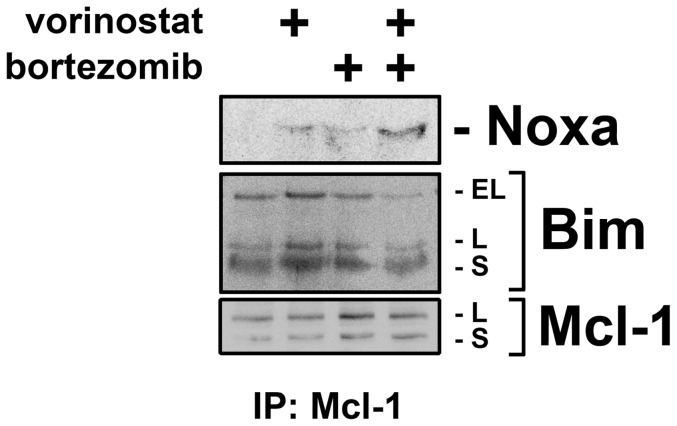
Noxa, when upregulated by vorinostat, binds to Mcl-1 and displaces pro-apoptotic Bim. M28 spheroids were treated with bortezomib (100 nM), vorinostat (5 µM) or their combination for 4 h, then studied by co-immunoprecipitation for Mcl-1 binding partners. Both vorinostat and bortezomib alone slightly increased the amount of Noxa bound to Mcl-1 but did not change the amount of bound Bim. However, the combination of vorinostat and bortezomib increased the amount of Noxa bound to Mcl-1 to a greater extent and reduced the amount of bound Bim. As expected, bortezomib increased total Mcl-1 slightly; nonetheless, the elevated Noxa was apparently sufficient to displace Bim.

Noxa has been shown to bind primarily to the anti-apoptotic protein Mcl-1, where it can displace Bim, thereby allowing Bim to mediate apoptosis by activating Bax and Bak [21]. To verify that the Noxa upregulated by vorinostat displaces pro-apoptotic Bim from Mcl-1, we performed a co-immunoprecipitation to measure the change in the amount of Noxa and Bim bound to Mcl-1 upon vorinostat (**Figure 4**). Following treatment with either vorinostat or bortezomib alone, there was a slight increase in the amount of Noxa bound to Mcl-1 but no apparent change in the binding of Bim. However, following the use of vorinostat together with bortezomib, there was an increased amount of Noxa bound to Mcl-1 and a reduced binding of Bim, confirming displacement of Bim.

**Figure 5:**
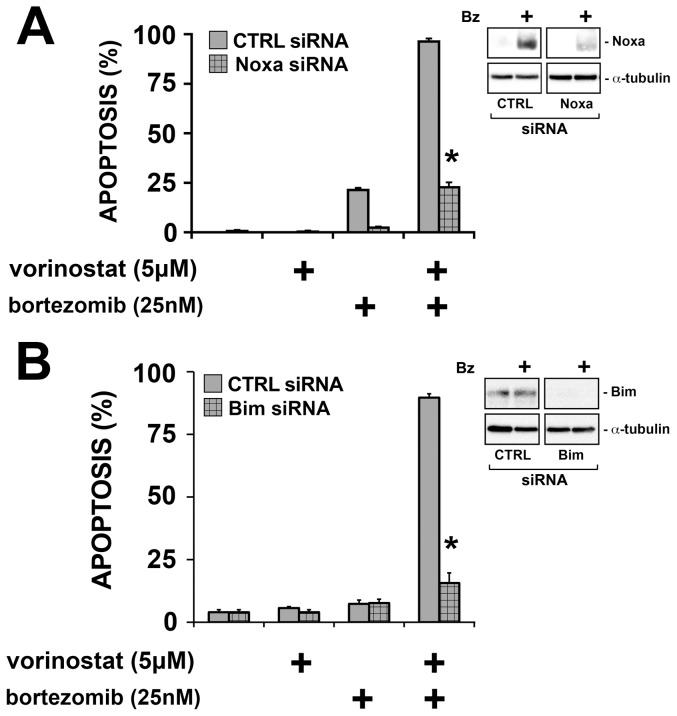
Noxa and Bim are each required for vorinostat to increase the apoptotic response of spheroids. REN cells were treated with siRNA to ablate Noxa or Bim, allowed to form spheroids over 24 h and then treated with bortezomib, vorinostat or the combination. Monolayers were treated similarly for 4 h and collected and lysed to verify the efficacy of Noxa siRNA (see immunoblots). After 24 h, Hoechst-stained cells were counted for the presence of apoptosis. Noxa and Bim siRNA significantly inhibited the ability of vorinostat to increase apoptosis in spheroids. (* p<0.05, significant difference compared to control siRNA; n = 3).

To confirm that the restored Noxa plays an essential role in the ability of vorinostat to reduce multicellular resistance, we ablated Noxa by siRNA before treating spheroids with bortezomib with or without vorinostat (**Figure 5**). In the absence of Noxa, vorinostat failed to increase bortezomib-induced apoptosis in 3D, thereby confirming a requirement for Noxa in the pro-apoptotic activity of vorinostat. We then asked whether Bim was also required for the activity of vorinostat. Indeed, in the absence of Bim, vorinostat also failed to enhance bortezomib-induced apoptosis in 3D. Thus, vorinostat acts via both Noxa and Bim in the elimination of multicellular resistance and the restoration of apoptosis in response to bortezomib.

### Vorinostat enhances the apoptotic response of mesothelioma cells in *ex vivo* tumor fragment spheroids

We then asked whether vorinostat would also increase chemoresponsiveness of mesothelioma cells within tumor fragment spheroids derived from human mesothelioma tissue. In this model, the mesothelioma cells reside in a complex tumor microenvironment [18] and represent a heterogeneous *ex vivo* tumor population.

**Figure 6:**
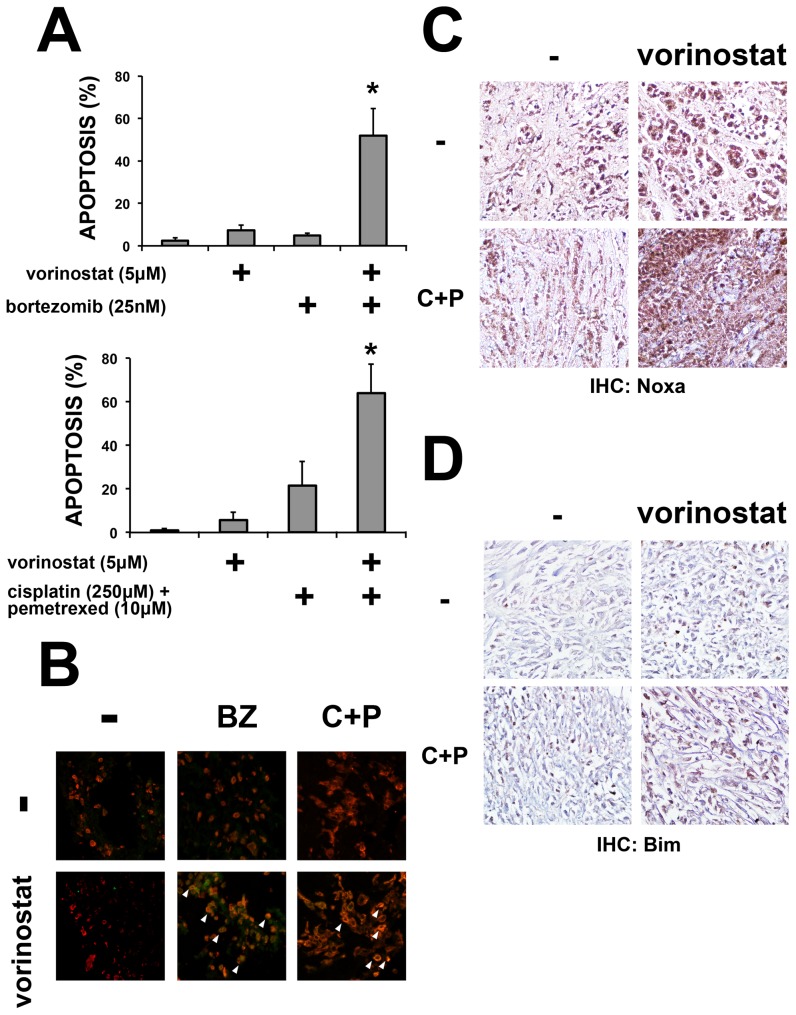
Vorinostat increases the apoptotic response of mesothelioma cells within tumor fragment spheroids to treatment. (**A**) Tumor fragment spheroids were grown from six treatment-naïve mesothelioma samples: three were treated with bortezomib (100 nM) and three with cisplatin (250 µM) plus pemetrexed (10 µM) (C+P) with or without vorinostat (5 µM) for 24 h. Apoptosis was measured by the detection of merged cytokeratin and cleaved caspase 3 immunofluorescence. In all samples, vorinostat increased the apoptotic response of mesothelioma cells to treatment. (* p<0.05 compared to chemotherapy or vorinostat alone, n = 3). (**B**) Representative confocal fluorescent merged images of tumor fragment spheroids after treatment. Addition of vorinostat either to bortezomib or to cisplatin plus pemetrexed increased the number of apoptotic mesothelioma cells (positive for both pan-cytokeratin and for cleaved caspase-3 staining). White arrowheads highlight some of the apoptotic cells. Images are representative of all tumor fragment spheroids used in the experiment. Immunohistochemistry for Noxa (**C**) and Bim (**D**) showed that the addition of vorinostat to treatment increases both Noxa and Bim in tumor fragment spheroids (images representative of the 3 tumor fragment spheroids treated with C+P in panel A).

We used tumor fragments from six different mesothelioma tumors and treated them with bortezomib (n = 3) or cisplatin plus pemetrexed (n = 3), with or without vorinostat. When used alone, vorinostat, bortezomib or cisplatin plus pemetrexed failed to induce a significant increase in apoptosis of mesothelioma cells. However, when vorinostat was added together with bortezomib or with cisplatin plus pemetrexed, mesothelioma cells in the tumor fragment spheroids from each tumor underwent a significant increase in apoptosis (**Figure 6A–B**). To assess whether vorinostat increased Noxa and Bim in the tissue, we stained the tumor fragment spheroids treated with vorinostat with or without chemotherapy. In the tumor fragment spheroids from all 3 tumors treated with vorinostat plus cisplatin and pemetrexed, Noxa and Bim protein expression increased (**Figure 6C–D**).

## Discussion

Solid tumors such as mesothelioma exhibit a resistance to chemotherapy that poses a challenge for curative therapy. We have attempted to model this chemoresistance in our 3D models, in which cells acquire multicellular resistance. Previously, we have reported that the acquired multicellular resistance is mediated by alterations in the Bcl-2 family of proteins, raising the hope that strategies directed to the Bcl-2 family could undermine resistance [1]. In this study, we have found that a broadly active histone deacetylase inhibitor, vorinostat, is able to ablate the multicellular resistance to bortezomib by its ability to restore expression of a key pro-apoptotic Bcl-2 family member, Noxa.

In our previous work in 3D spheroids, resistance to bortezomib was shown to be due to a lack of upregulation of Noxa, a sensitizer BH3-only protein that acts via displacement of pro-apoptotic Bim from Mcl-1 and possibly also from Bcl-2 and Bcl-xl [1], [22], [23]. Importantly, we found that spheroids expressed elevated Bim and were primed for apoptosis but, without Noxa to displace the Bim from anti-apoptotic buffers, the spheroids exhibited a resistance to apoptosis. Therefore, in this study, we sought means of restoring the upregulation of Noxa in the spheroids and turned to vorinostat, an HDAC inhibitor thought to act via enhanced transcription and expression of epigenetically-silenced genes, many of which are pro-apoptotic [24]. Indeed, vorinostat effectively restored the pro-apoptotic Noxa upregulation seen in response to bortezomib. By RNA silencing, Noxa and Bim were each shown to be required for the ability of vorinostat to undermine multicellular resistance. Vorinostat appeared to act at least in part via the transcriptional upregulation of Noxa; in 3D, the increase in Noxa message in response to bortezomib was blunted and, with the addition of vorinostat, the Noxa message was restored to the same levels seen in monolayers. In addition to its beneficial effect on Noxa regulation, vorinostat increased baseline levels of Bim protein, in both multicellular spheroids and tumor fragment spheroids, suggesting that vorinostat could perhaps elevate Bim in tumors with low Bim, found to represent approximately 30% of mesotheliomas [1].

Our findings may have clinical relevance for the use of HDAC inhibitors in combinatorial therapy. Despite promising *in vitro* and pre-clinical results, vorinostat and bortezomib have each been found to be largely ineffective as single agents in clinical trials of patients with relapsed mesothelioma [*unpublished*, http://goo.gl/dZCFx] [9]. Our results in 3D spheroids are similar: neither agent was effective in inducing apoptosis in 3D spheroids when given alone. In particular, vorinostat alone had almost no apparent effect against mesothelioma cells in 3D spheroids; there was no evident impact of vorinostat alone on cellular morphology, Noxa transcription, Noxa protein levels or apoptosis. In our models, the benefit of vorinostat was only seen when it was used with other agents. Indeed, in clinical trials, whereas vorinostat as a single agent has yielded disappointing results, its use in combination with chemotherapy has been reported to have positive results in a variety of solid and hematological malignancies [25]. It is of interest then that, despite the lack of benefit reported for vorinostat as a single agent in mesothelioma, another HDAC inhibitor, valproic acid, was recently found to have benefit when used with doxorubicin in mesothelioma patients [26]. Our findings in mesothelioma lead us to believe that the key to clinical efficacy of HDAC inhibition lies in its ability to potentiate responses to chemotherapy [27], a potentially valuable strategy against chemoresistant solid tumors. Recently, it has been reported that resistance to manipulation of Noxa can develop over a period of 10 days [28]. Although we did not study our spheroids for that duration, we saw no resistance to vorinostat over 2 days (data not shown). Issues including drug resistance as well as drug penetrance and toxicity will be important considerations in the use of this combination in the clinic [29].

In summary, we have used 3D models to show the value of histone deacetylase inhibition in undermining multicellular resistance to chemotherapy. This raises the possibility that the resistance of the tumor cells in 3D, which can be localized to the Bcl-2 family, is mediated via epigenetic mechanisms. In the 3D resistant setting, chemoresponsiveness can be enhanced by either targeting the Bcl-2 family directly, as we have previously done by using small molecules such as ABT-737 [1] or indirectly, by restoring transcription of silenced pro-apoptotic genes, as we show here. Possibly both strategies could be used together, especially if vorinostat enhances Bim expression in tumors with low baseline expression. These studies may provide the basis for the use of these agents in combination in future clinical trials.

## Supporting Information

Figure S1 **Vorinostat increases apoptotic response of spheroids to cisplatin plus pemetrexed.** M28 and REN spheroids were treated with cisplatin (250 µM) plus pemetrexed (10 µM), vorinostat (5 µM) or their combination for 24 h. Apoptosis was measured by CaspaseGlo 3/7 assay. Cisplatin plus pemetrexed (C+P) or vorinostat alone had no effect on spheroids but the combination induced a significant apoptotic response (* p<0.05 as compared to cisplatin plus pemetrexed or vorinostat alone, n = 3)
(TIFF)

Figure S2 **Densitometry analysis for bands shown in the western blot in** **Figure 2** **: M28 (A) and REN (B).** Intensity of each band was determined by densitometry and expressed relative to the intensity of the corresponding alpha-tubulin band.
(TIFF)

Figure S3 **Vorinostat increases the levels of Noxa and Bim induced by cisplatin plus pemetrexed in spheroids.** M28 and REN spheroids were treated with cisplatin (250 µM) plus pemetrexed (10 µM) (C+P) (alone or with vorinostat (5 µM)) for 6 h. Cells were then lysed and Noxa and Bim levels were analyzed by western-blot. The addition of vorinostat increased the Noxa and Bim response to cisplatin plus pemetrexed.
(TIFF)

Figure S4 **Vorinostat in combination with bortezomib increases Noxa message more than that of Bim or Bmf.** M28 and REN spheroids were treated with bortezomib (100 nM) plus vorinostat (5 µM) for 4 h. Noxa, Bim and Bmf mRNA levels were determined by qRT-PCR. Noxa mRNA levels increased more than that of Bim or Bmf.
(TIFF)
