# Supplementary figures and images for: Vorinostat Eliminates Multicellular Resistance of Mesothelioma 3D Spheroids via Restoration of Noxa Expression
Source: PLoS One. 2012 Dec 26;7(12):e52753. doi: 10.1371/journal.pone.0052753 (PMC3530471; doi:10.1371/journal.pone.0052753)

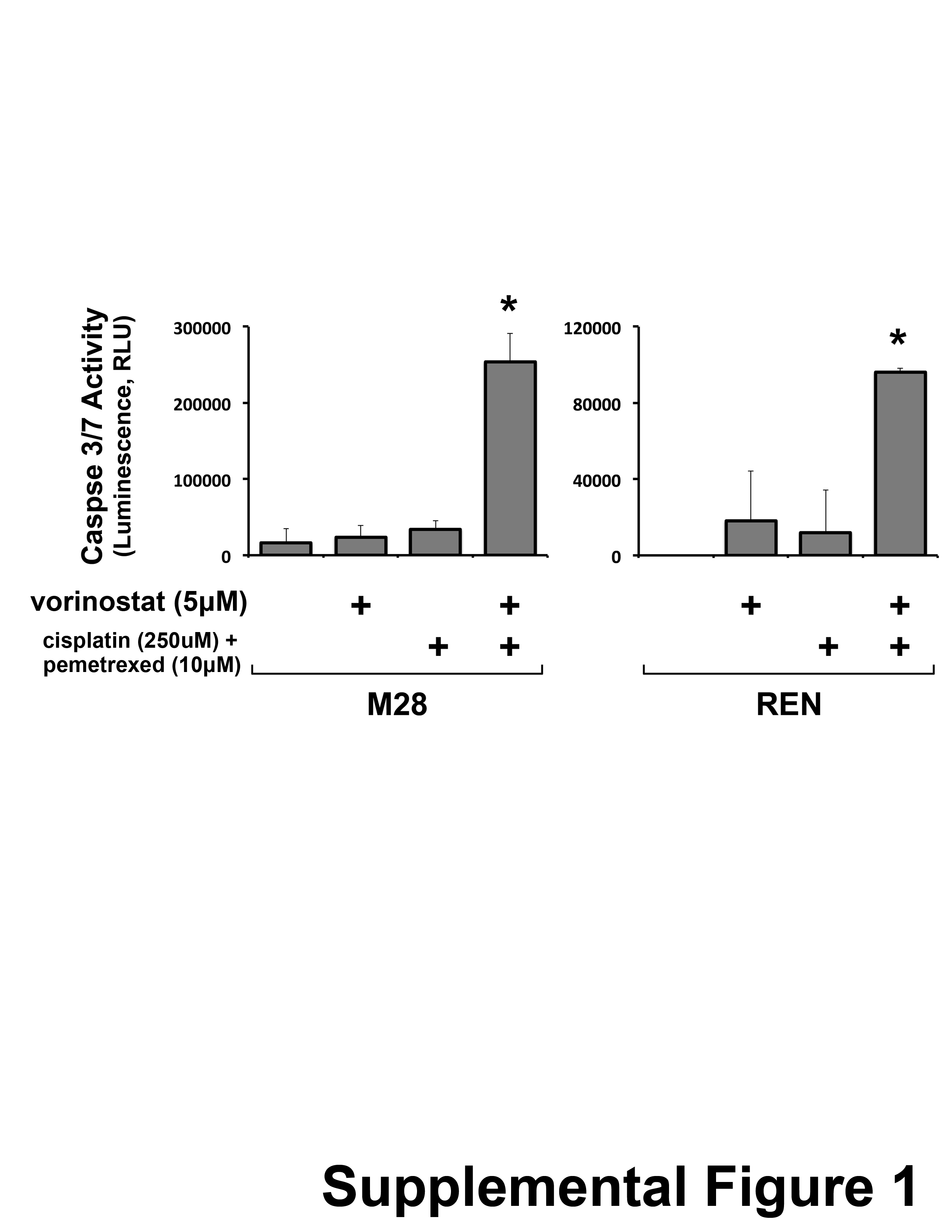

Supplement: Figure S1 — Vorinostat increases apoptotic response of spheroids to cisplatin plus pemetrexed. M28 and REN spheroids were treated with cisplatin (250 µM) plus pemetrexed (10 µM), vorinostat (5 µM) or their combination for 24 h. Apoptosis was measured by CaspaseGlo 3/7 assay. Cisplatin plus pemetrexed (C+P) or vorinostat alone had no effect on spheroids but the combination induced a significant apoptotic response (* p<0.05 as compared to cisplatin plus pemetrexed or vorinostat alone, n = 3) (TIFF) [file pone.0052753.s001.tiff]

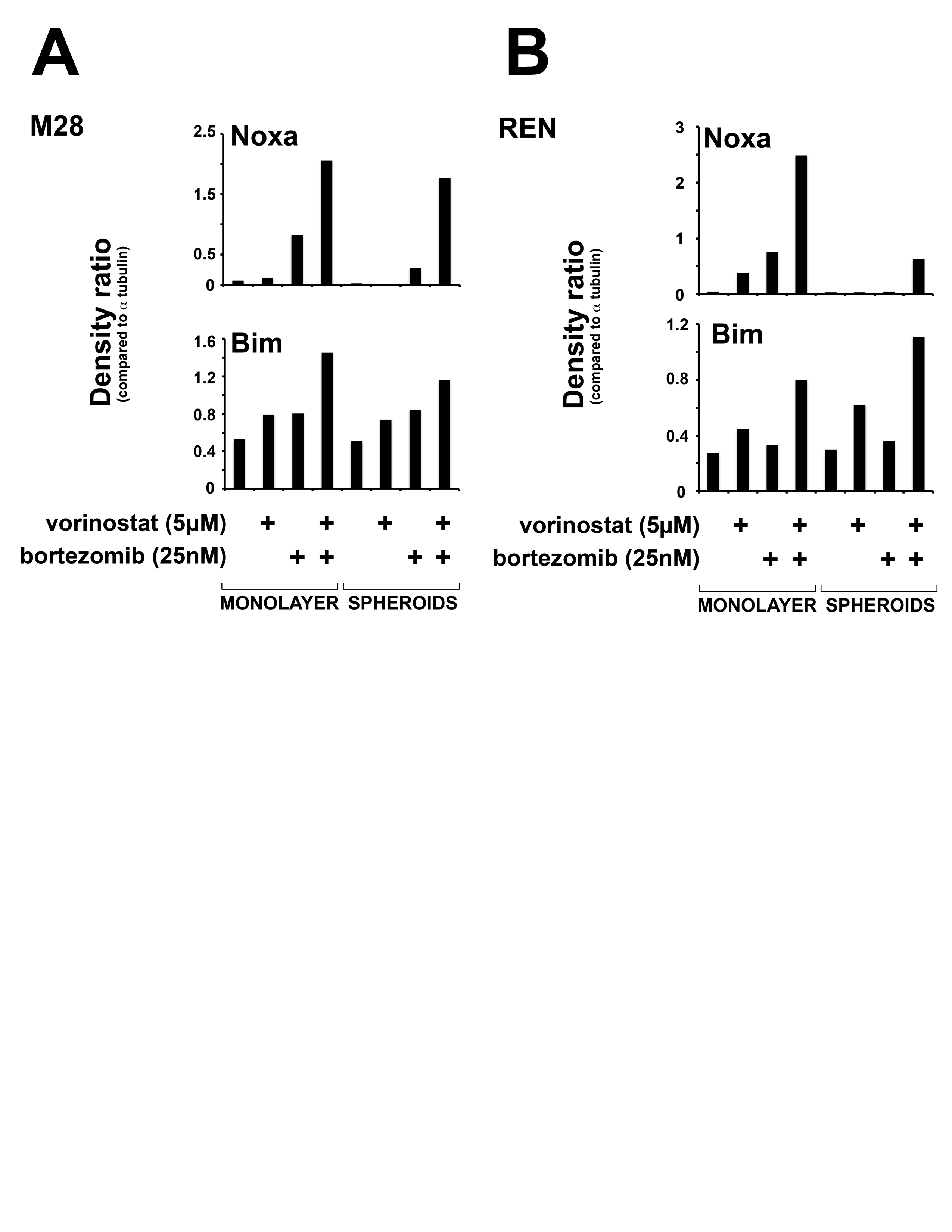

Supplement: Figure S2 — Densitometry analysis for bands shown in the western blot in Figure 2 : M28 (A) and REN (B). Intensity of each band was determined by densitometry and expressed relative to the intensity of the corresponding alpha-tubulin band. (TIFF) [file pone.0052753.s002.tiff]

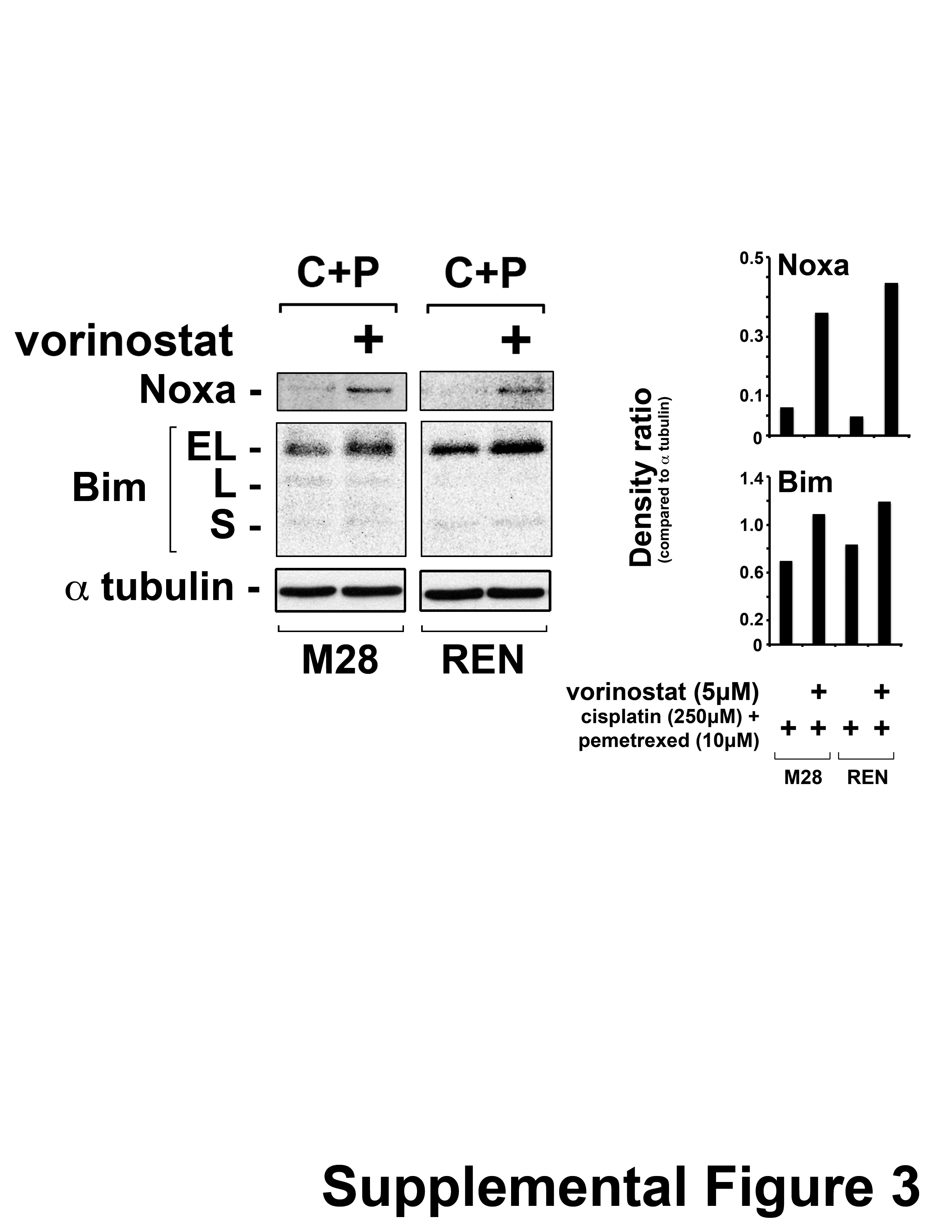

Supplement: Figure S3 — Vorinostat increases the levels of Noxa and Bim induced by cisplatin plus pemetrexed in spheroids. M28 and REN spheroids were treated with cisplatin (250 µM) plus pemetrexed (10 µM) (C+P) (alone or with vorinostat (5 µM)) for 6 h. Cells were then lysed and Noxa and Bim levels were analyzed by western-blot. The addition of vorinostat increased the Noxa and Bim response to cisplatin plus pemetrexed. (TIFF) [file pone.0052753.s003.tiff]

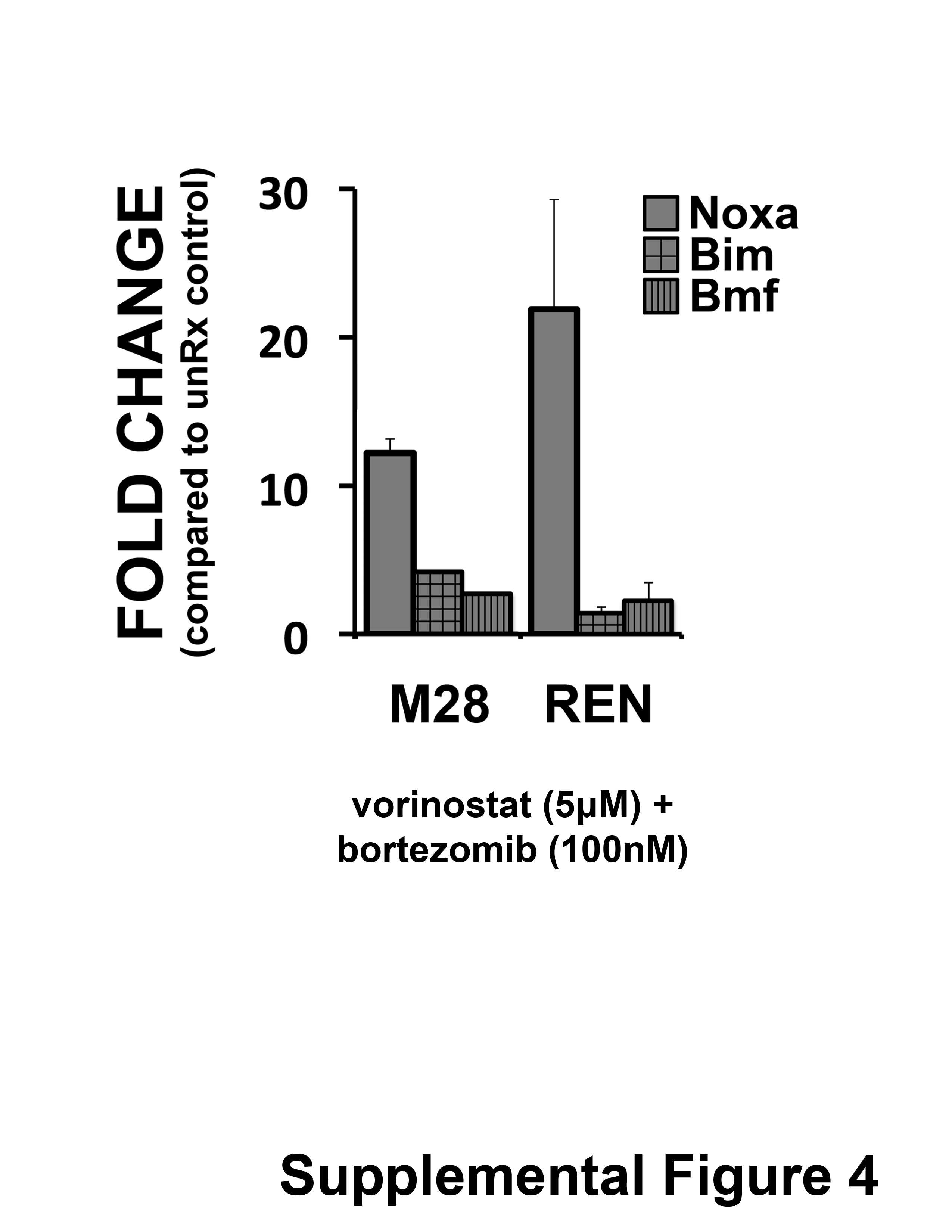

Supplement: Figure S4 — Vorinostat in combination with bortezomib increases Noxa message more than that of Bim or Bmf. M28 and REN spheroids were treated with bortezomib (100 nM) plus vorinostat (5 µM) for 4 h. Noxa, Bim and Bmf mRNA levels were determined by qRT-PCR. Noxa mRNA levels increased more than that of Bim or Bmf. (TIFF) [file pone.0052753.s004.tiff]
